# Assessing the importance of predictors of adherence to a digital self‑management intervention for osteoarthritis

**DOI:** 10.1186/s13018-023-03562-6

**Published:** 2023-02-13

**Authors:** Ali Kiadaliri, Andrea Dell’Isola, L. Stefan Lohmander, David J. Hunter, Leif E. Dahlberg

**Affiliations:** 1 Clinical Epidemiology Unit, Department of Clinical Sciences Lund, Orthopedics, Lund University, Lund, Sweden; 2 Centre for Economic Demography, Lund University, Lund, Sweden; 3 Arthro Therapeutics, Malmö, Sweden; 4 Department of Clinical Sciences Lund, Orthopedics, Lund University, Lund, Sweden; 5 Sydney Musculoskeletal Health, Kolling Institute of Medical Research, University of Sydney, Sydney, Australia; 6 Clinical Epidemiology Unit, Skåne University Hospital, Remissgatan 4, 221 85 Lund, Sweden

**Keywords:** Adherence, Digital therapy, Dominance analysis, Osteoarthritis, Self-management, Sweden

## Abstract

**Objective:**

Treatment adherence is suggested to be associated with greater improvement in patient outcomes. Despite the growing use of digital therapeutics in osteoarthritis management, there is limited evidence of person-level factors influencing adherence to these interventions in real-world settings. We aimed to determine the relative importance of factors influencing adherence to a digital self-management intervention for hip/knee osteoarthritis.

**Methods:**

We obtained data from people participating in a digital OA treatment, known as Joint Academy, between January 2019 and September 2021. We collected data on the participants’ adherence, defined as the percentage of completed activities (exercises, lessons, and quizzes), at 3 (*n* = 14,610)- and 12-month (*n* = 2682) follow-up. We used dominance and relative weight analyses to assess the relative importance of sociodemographic (age, sex, place of residence, education, year of enrolment), lifestyle (body mass index, physical activity), general health (comorbidity, overall health, activity impairment, anxiety/depression), and osteoarthritis-related (index joint, fear of moving, walking difficulties, pain, physical function, wish for surgery, Patient Acceptable Symptom State) factors, measured at baseline, in explaining variations in adherence. We used bootstrap (1000 replications) to compute 95% confidence intervals.

**Results:**

Mean (SD) adherences at 3 and 12 months were 86.3% (16.1) and 84.1% (16.7), with 75.1% and 70.4% of participants reporting an adherence ≥ 80%, respectively. The predictors included in the study explained only 5.6% (95% CI 5.1, 6.6) and 8.1% (7.3, 11.6) of variations in 3- and 12-month adherences, respectively. Sociodemographic factors were the most important predictors explaining more variations than other factors altogether. Among single factors, age with a nonlinear relationship with adherence, was the most important predictor explaining 2.3% (95% CI 1.9, 2.8) and 3.7% (2.4, 5.3) of variations in 3- and 12-month adherences, respectively.

**Conclusion:**

Person-level factors could only modestly explain the variations in adherence with sociodemographic characteristics, mainly age, accounting for the greatest portion of this explained variance.

**Supplementary Information:**

The online version contains supplementary material available at 10.1186/s13018-023-03562-6.

## Introduction

Osteoarthritis (OA), the most common joint disorder, is a disabling condition characterized by pain, stiffness, loss of function, and impaired quality of life [1–3]. With no cure for OA, patient education, self-management, exercises, and weight control are recommended as core treatments [4]. However, implementation of these recommendations in clinical practice is suboptimal, with two recent systematic reviews reporting that only one in three persons with OA receives appropriate non-pharmacological care according to the guidelines [5, 6]. Lack of knowledge about treatment approaches and guidelines by healthcare providers and patients, limited provision of these treatments, and constraints related to access, time, and costs have been identified as major barriers to implementing these recommendations in clinical practice [7–9].

Digital therapeutics, including smartphone apps, have emerged as an accessible solution to address these barriers with the potential to facilitate better patient-provider interactions and lead to more personalized care [7, 10, 11]. These interventions have shown efficacy in promoting physical activity and improving patients’ pain, function, and quality of life [7, 12, 13]. A major requisite to a digital intervention’s success is adherence [14, 15], defined as the extent to which a person’s behaviour matches agreed recommendations [16]. While clinical trials generally report relatively high adherence to digital self-management interventions for OA, these have mostly short study durations and may not reflect routine practices in real-world settings [10]. Research on adherence, including person-level factors influencing adherence in real-world settings, is very limited [15]. Better insight into factors influencing adherence may help to identify participants who benefit most from digital interventions, and to develop more targeted solutions for those at a higher risk of not adhering to these interventions. Previous research has mainly focused on average associations (e.g. regression coefficients, odds ratios) and statistical significance to identify the predictors of adherence. Such analyses are unable to provide information on the predictive ability of predictors and their relative importance in predicting an outcome [17]. We here explored the relative importance of person-level factors in adherence to a digital platform for OA self-management.

## Methods

### Study design and data source

This was an observational registry-based study using data extracted from the Joint Academy® registry in February 2022 (https://www.jointacademy.com/us/en/). The registry data are routinely collected through participants’ use of the Joint Academy application. Participants in the digital programme either had a prior radiographic and/or clinical diagnosis of hip or knee OA from a physical therapist or physician or were, after responding to a series of diagnostic inclusion/exclusion questions, confirmed to have clinical OA by a physiotherapist via phone [18]. If the diagnosis remained uncertain, participants were referred for a physical visit.

### Intervention

Joint Academy® (JA) is a digital self-management programme for OA targeted towards exercise, physical activity and education and delivered by a smartphone application. It was introduced in Sweden in 2016 [19] as a digital version of the evidence-based structured first-line face-to-face self-management programme, known as “Better management of patients with OsteoArthritis” [20]. The digital programme contains video lectures on OA, physical activity, and self-management (a total of 70 lectures over a 48-week period) as well as individualized neuromuscular exercises, generally two per day during the whole participation period, with complexity and difficulty levels that are adjusted to each participant’s progression in the programme. The participants are supervised by their physical therapist through a therapist-specific app during the full participation period and are able to chat asynchronously with their personal therapist for feedback and questions [18, 19]. The programme is covered by the national healthcare system in Sweden.

### Participants

We included all consecutive participants with hip or knee OA enrolled in the digital programme between January 1st, 2019 and September 30th, 2021, who provided their informed consent for research at enrolment (*n* = 16,640). Of these, we excluded 1912 (11.5%) who stopped using the app prior to the 3-month follow-up, and 118 (0.7%) with missing records of adherence.

### Measures

#### Adherence to digital programme

Adherence was defined as the percentage (0–100) of completed activities (exercises, text or video lessons, and quizzes on lesson material) over 3 (12 weeks) and 12 months (48 weeks) of participation in the intervention. These were measured as the average of weekly completed activities. In addition, we defined, as a secondary outcome, “low adherence” as adherence of less than 80%. This threshold was chosen following the Swedish clinical practice guideline recommendations for first-line treatments of knee and hip OA [21]. The 80% threshold is believed to be, and the most commonly used, level to determine satisfactory adherence to therapeutic exercise for musculoskeletal pain [22].

#### Predictors

We grouped the factors influencing adherence into four categories: sociodemographic characteristics, lifestyle factors, general health-related factors, and OA-related factors. All predictors were self-reported and measured at baseline.

Sociodemographic characteristics included age, sex, year of enrolment, education (less than high school, high school, college/university degree), and place of residence (living in three largest Swedish cities [Stockholm/Gothenburg/Malmö], living in other places, unknown/missing place of residence). Lifestyle factors comprised body mass index (BMI) and physical activity. BMI was categorized into four groups: normal (BMI < 25), overweight (25 ≤ BMI < 30), obese (30 ≤ BMI < 35) and morbidly obese (BMI ≥ 35). Physical activity was measured based on the responses to the following question: “How much time do you spend in a typical week on daily physical activity that is not exercise, such as walking, cycling or gardening?” [23]. Responses were coded into six categories: less than 30 min, 30–60 min, 61–90 min, 91–150 min, 151–300 min, and more than 300 min.

General health-related factors included comorbidity, overall health, activity impairment, and anxiety/depression. The following self-reported doctor diagnosed comorbidities were included: diabetes, lung diseases, balance troubles, rheumatoid arthritis, cardiovascular diseases, and pain in other joints. Overall health was measured using a 11-point numerical rating scale (NRS, “Mark on the scale how good or bad your current health is?” with 0 = worst imaginable and 10 = best imaginable). Activity impairment was measured using a 11-point NRS (“During the past 7 days, how much did knee/hip osteoarthritis affect your ability to do your regular daily activities, other than work at a job?”, with 0 = it had no effect on my daily activities and 10 = it completely prevented me from doing my daily activities) from the Work Productivity and Activity Impairment–Osteoarthritis (WPAI–OA) questionnaire [24]. Anxiety/depression was measured using the responses to EQ-5D-5L anxiety/depression dimension (no problem, slight problems, moderate problems, and severe problems).

OA-related factors included index joint (the most painful joint: hip or knee), fear of moving (yes/no), walking difficulties (yes/no), pain, physical function, wish for surgery, and Patient Acceptable Symptom State (PASS). Pain was assessed using an 11-point NRS (0 = no pain and 10 = the worst possible pain). Physical function was measured using the 30-s chair stand test (30CST), in which the participants sit and stand from a chair for 30 s and reported the maximum number of repetitions. Wish for surgery and PASS were recorded by the participants’ “Yes” or “No” answers to the following questions, respectively: “Are your symptoms so severe that you wish to undergo surgery in your knee/hip?”, and “Considering your hip/knee function, do you feel that your current state is satisfactory? With hip/knee function, you should take into account all activities you have during your daily life, sport and recreational activities, your level of pain and other symptoms, and quality of life.” [25].

### Data analysis

We employed dominance analysis to assess the importance of grouped predictors. Dominance analysis quantifies the contribution of each predictor to the change in model fit statistics across all possible submodels for a given set of predictors, excluding constant-only model (*k* predictors correspond to 2^*k *^− 1 submodels) [26, 27]. We used linear regression and *R*^2^ as fit statistics for analysing adherence. For low adherence (adherence < 80%), we utilized logistic regression and the McFadden pseudo-*R*^2^ as fit statistics. These fit statistics can be interpreted as the proportion of total variance in adherence which can be explained by the predictors included in the study. From dominance analysis, we reported general dominance statistics, which reflect a predictor’s average incremental contribution to R^2^/McFadden pseudo-*R*^2^ across all possible submodels. An appealing feature of general dominance statistics is that the sum of general dominance for predictors is equal to the full model fit statistics [17]. This implies that the relative contribution of a predictor can be calculated by dividing its general dominance statistics on the full model *R*^2^/McFadden pseudo-*R*^2^.

As a secondary analysis, we computed the relative contribution of every single predictor using relative weight analysis [28]. The reason to use relative weight analysis in place of dominance analysis was that while it produces largely similar results to dominance analysis [29], it is much faster computationally (using dominance analysis for 18 predictors in our study with 1000 bootstrapped replications would require over 260 million submodels which could take days to run). Dominance and relative weight analyses were conducted using Stata’s “domin” command [30]. We inspected the linearity of the continuous variables using residual plots and modelled nonlinear relationships using linear splines. We used bootstrapping with 1000 replications to calculate 95% confidence intervals (CI).

## Results

A total of 14,610 persons, mean (SD) age 64.1 (9.1) and 75.5% females, with data on adherence at 3-month follow-up were included (Table 1). Of these, 2682 persons had data on adherence at 12-month follow-up. These two groups had comparable baseline characteristics, with the main exception being the year of enrolment, which was expected (most persons enrolled in 2021 did not have 12-month follow-up due to the study time frame). We also excluded 2030 individuals with no follow-up responses. Compared to those included in the study, these persons were more likely to not report their place of residence, were less physically active, and had more comorbidities and anxiety/depression, as well as a larger proportion of wish for surgery at baseline.

**Table 1:** Baseline characteristics of participants in the digital intervention

	Included		Excluded
	3-month follow-up	12-month follow-up	
*N*	14,610	2682	2030
Female, *n* (%)	11,029 (75.5)	2044 (76.2)	1532 (75.5)
Age, mean (SD)	64.1 (9.1)	64.3 (8.6)	65.6 (10.3)
Enrolment year, *n* (%)			
2019	925 (6.3)	348 (13.0)	382 (18.8)
2020	3666 (25.1)	1850 (69.0)	532 (26.2)
2021	10,019 (68.6)	484 (18.0)	1116 (55.0)
Place of residence, *n* (%)			
Stockholm/Gothenburg/Malmö	2765 (18.9)	534 (19.9)	387 (19.1)
Other locations	11,593 (79.4)	2139 (79.8)	1342 (66.1)
Unknown/missing	252 (1.7)	9 (0.3)	301 (14.8)
Education, *n* (%)			
Less than high school	1181 (8.1)	183 (6.8)	183 (9.0)
High school	5244 (35.9)	872 (32.5)	719 (35.4)
College/university	8185 (56.0)	1627 (60.7)	1128 (55.6)
Body mass index, mean (SD)	27.2 (4.7)	27.0 (4.8)	27.2 (4.8)
Physical activity, *n* (%)			
Less than 30 min	1017 (6.7)	150 (5.6)	185 (9.1)
30–60 min	2288 (15.7)	397 (14.8)	382 (18.8)
61–90 min	2182 (14.9)	372 (13.9)	288 (14.2)
91–150 min	2601 (17.8)	466 (17.4)	353 (17.4)
151–300 min	3358 (23.0)	649 (24.2)	427 (21.0)
More than 300 min	3164 (21.7)	648 (24.2)	395 (19.5)
Pain in other joints, *n* (%)	11,215 (76.8)	2079 (77.5)	1575 (77.6)
Diabetes, *n* (%)	819 (5.6)	157 (5.9)	174 (8.6)
Lung diseases, *n* (%)	1556 (10.7)	289 (10.8)	230 (11.3)
Balance troubles, *n* (%)	483 (3.3)	102 (3.8)	117 (5.8)
Rheumatoid arthritis, *n* (%)	662 (4.5)	126 (4.7)	135 (6.7)
Cardiovascular diseases, *n* (%)	1078 (7.4)	184 (6.9)	216 (10.6)
Overall health (NRS, 0–10), mean (SD)	6.6 (1.8)	6.6 (1.9)	6.4 (2.0)
Activity impairment (NRS, 0–10), mean (SD)	3.9 (2.4)	3.9 (2.4)	4.2 (2.5)
EQ-5D-5L anxiety/depression, *n* (%)			
No problem	7192 (49.2)	1276 (47.6)	956 (47.1)
Slight problems	5634 (38.6)	1072 (40.0)	759 (37.4)
Moderate problems	1451 (9.9)	272 (10.1)	241 (11.9)
Severe problems	333 (2.3)	62 (2.3)	74 (3.7)
Index joint, *n* (%)			
Knee	8758 (60.0)	1662 (62.0)	1080 (53.2)
Hip	5852 (40.0)	1020 (38.0)	950 (46.8)
Pain (NRS, 0–10), mean (SD)	5.1 (1.9)	5.0 (1.9)	5.2 (2.5)
Physical function, mean (SD)	12.8 (4.3)	11.6 (4.0)	12.0 (4.4)
Walking difficulties, *n* (%)	9585 (65.6)	1764 (65.8)	1392 (68.6)
Fear of moving, *n* (%)	2205 (15.1)	408 (15.2)	295 (14.5)
Wish for surgery, *n* (%)	2296 (15.7)	386 (14.4)	413 (20.3)
Patient Acceptable Symptom State, *n* (%)	2459 (16.8)	412 (15.4)	346 (17.0)

*NRS* numerical rating scale

Mean (SD) adherence at the 3-month follow-up (*n* = 14,610) was 86.3% (16.1), with 24.9% of participants having low adherence. The corresponding figures at 12-month follow-up (*n* = 2682) were 84.1% (16.7) and 29.6%, respectively. The predictors included in this study explained 5.6% (95% CI 5.1, 6.6) and 8.1% (7.3, 11.6) of variations in adherence at 3- and 12-month follow-ups, respectively (Table 2). Dominance analysis revealed that sociodemographic characteristics were the most important predictors explaining 3.3% (95% CI 2.8, 3.9) and 5.4% (4.0, 7.4) of variations in 3- and 12-month adherence, corresponding to 59.1% (95% CI 51.0, 63.9) and 66.3% (49.9, 70.4) relative contributions, respectively. Despite the differences in point estimates of the contributions of other factors, the bootstrapped 95% CI of their differences were wide and inconclusive (i.e. included 0), suggesting comparable contributions of other factors to adherence at both time points (Table 3). Using low adherence as the outcome generally yielded similar results with two main exceptions: the general health-related factors had more important contributions than OA-related factors at 3-month follow-up and the rank order of factors other than sociodemographic factors changed for 12-month follow-up.

**Table 2:** Absolute and relative contributions of person-level factors to adherence to the digital intervention

	3-month adherence			12-month adherence		
	General dominance statistics	Relative contribution	Rank^a^	General dominance statistics	Relative contribution	Rank^a^
*Adherence*						
Sociodemographic characteristics	0.033 (0.028, 0.039)	59.1 (51.0, 63.9)	1	0.054 (0.040, 0.074)	66.3 (49.9, 70.4)	1
Lifestyle factors	0.008 (0.006, 0.012)	15.0 (10.6, 19.9)	3	0.011 (0.007, 0.022)	13.2 (7.5, 24.1)	3
General health-related factors	0.009 (0.007, 0.013)	15.3 (11.7, 20.9)	2	0.008 (0.006, 0.020)	10.2 (6.6, 21.1)	3
Osteoarthritis-related factors	0.006 (0.004, 0.009)	10.6 (7.5, 15.2)	4	0.008 (0.006, 0.019)	10.2 (6.4, 19.1)	3
Overall *R*^2^	0.056 (0.051, 0.066)			0.081 (0.073, 0.116)		
*Low adherence (adherence < 80%)*						
Sociodemographic characteristics	0.019 (0.016, 0.024)	52.7 (43.3, 58.3)	1	0.034 (0.026, 0.050)	63.8 (44.3, 68.8)	1
Lifestyle factors	0.006 (0.005, 0.009)	17.4 (12.5, 23.5)	3	0.005 (0.003, 0.013)	9.0 (5.0, 20.5)	4
General health-related factors	0.007 (0.006, 0.011)	20.4 (15.7, 27.3)	2	0.008 (0.006, 0.018)	14.1 (9.1, 27.2)	2
Osteoarthritis-related factors	0.003 (0.002, 0.006)	9.5 (6.5, 14.8)	4	0.007 (0.005, 0.016)	13.1 (7.8, 24.0)	3
Overall McFadden pseudo-*R*^2^	0.036 (0.033, 0.044)			0.054 (0.050, 0.082)		

^a^Based on median rank in 1000 bootstrapped replications

**Table 3:** Mean differences (95% CI) in general dominance statistics of adherence predictors

	3-month adherence			12-month adherence		
	Sociodemographic versus	Lifestyle versus	General health versus	Sociodemographic versus	Lifestyle versus	General health versus
*Adherence*						
Sociodemographic	–	–	–	–	–	–
Lifestyle	0.025 (0.018, 0.031)	–	–	0.043 (0.024, 0.062)	–	–
General health	0.024 (0.018, 0.031)	− 0.0002 (− 0.005, 0.004)	–	0.046 (0.027, 0.062)	0.002 (− 0.010, 0.013)	–
Osteoarthritis	0.027 (0.021, 0.034)	0.002 (− 0.001, 0.006)	0.003 (− 0.001, 0.007)	0.046 (0.028, 0.063)	0.002 (− 0.007, 0.013)	0.000 (− 0.009, 0.011)
*Low adherence*						
Sociodemographic	–	–	–	–	–	–
Lifestyle	0.013 (0.008, 0.017)	–	–	0.029 (0.016, 0.044)	–	–
General health	0.012 (0.007, 0.016)	− 0.001 (− 0.005, 0.002)	–	0.027 (0.012, 0.040)	− 0.003 (− 0.012, 0.005)	–
Osteoarthritis	0.016 (0.011, 0.020)	0.003 (− 0.000, 0.006)	0.004 (0.001, 0.007)	0.027 (0.014, 0.042)	− 0.002 (− 0.010, 0.005)	0.001 (− 0.007, 0.009)

**Fig. 1:**
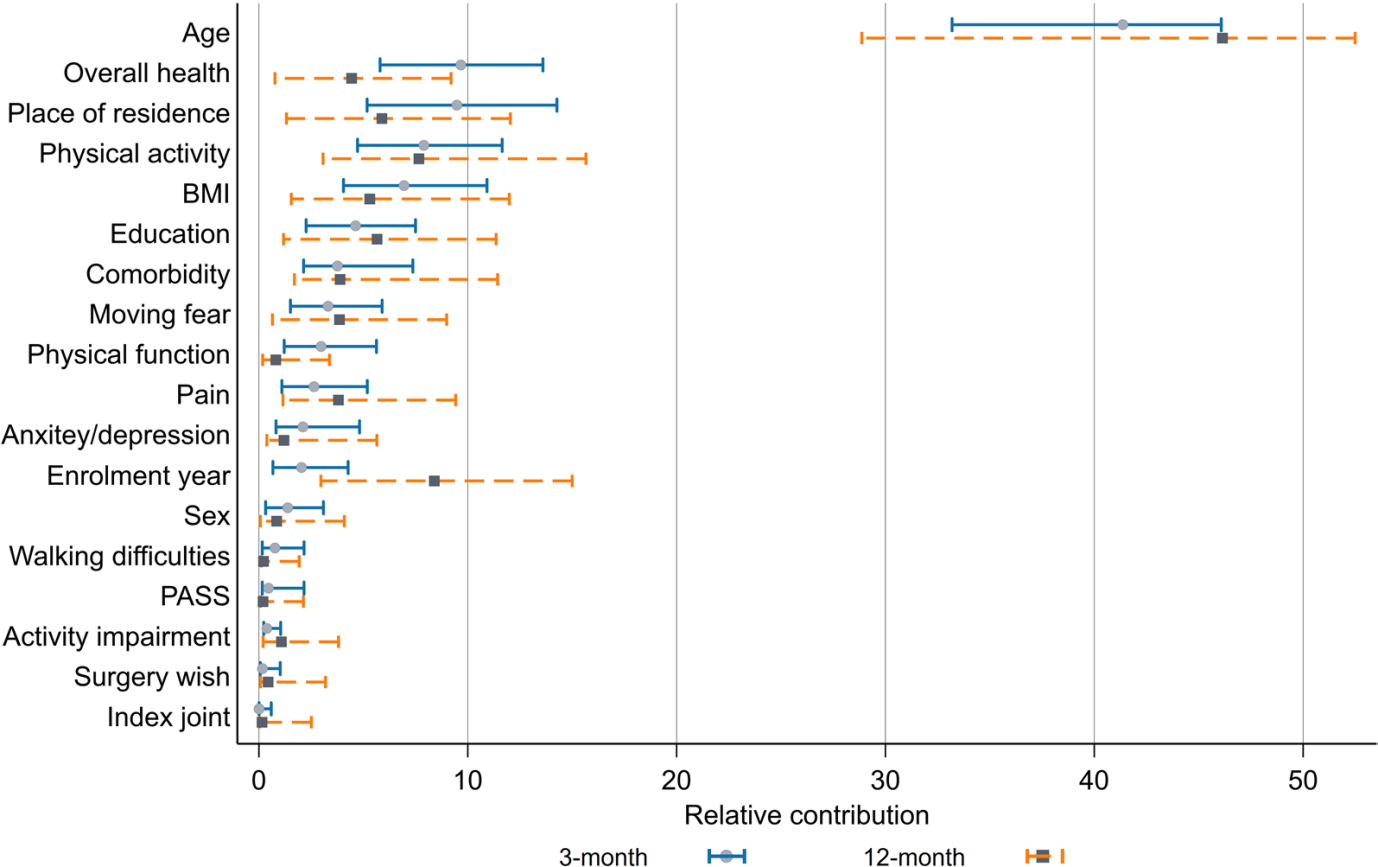
The relative contributions of predictors to the explained variance of adherence at 3- and 12-month follow-ups estimated using relative weight analysis

**Fig. 2:**
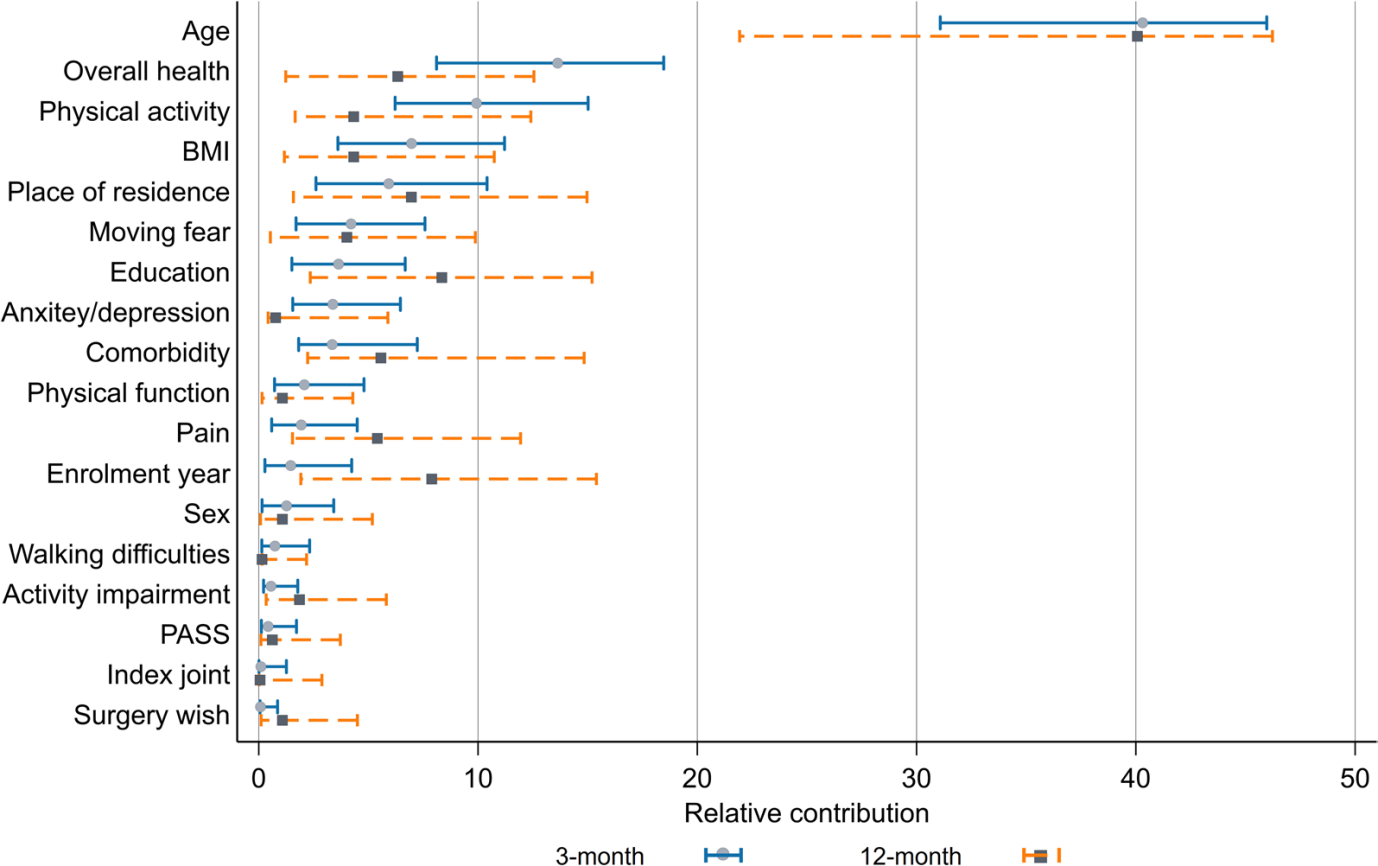
The relative contributions of predictors to the explained variance of low adherence (adherence < 80%) at 3- and 12-month follow-ups estimated using relative weight analysis

The associations between adherence and single predictors suggested that adherence was generally positively associated with female sex, enrolment in more recent years, living outside three largest cities, lower education, having BMI < 25, higher physical activity, having walking difficulties, no fear of moving, no comorbidity, no anxiety/depression, and not being satisfied with symptoms (Additional file 1: Tables S1–S4). The associations between adherence and age, pain, overall health as well as physical function were generally nonlinear (e.g. 1-year increase in age was associated with 0.3% increase up to age 75 years and 0.5% reduction in ages older than 75 years in 3-month adherence). Among these single predictors, age was by far the most important predictor in both time points, accounting for over 40% of the explained variance in adherence/low adherence (Figs. 1, 2). For 3-month adherence, overall health and place of residence ranked as 2nd and 3rd important predictors, for 12-month adherence the year of enrolment in the programme and physical activity had these positions. For low adherence at 3 months, overall health and physical activity were the most important predictors following age, while education and the year of enrolment in the programme were the most important ones at 12 months. Among OA-related factors, fear of moving was the most important predictor of adherence, followed by pain and physical function.

## Discussion

This study is the first to apply dominance and relative weight analyses to investigate the relative importance of person-level factors in predicting adherence to a digital self-management programme for OA. Our results suggested that the broad array of person-level factors included in the study could explain only a modest amount (4–8%) of variations in adherence. Sociodemographic characteristics were the most important predictors of adherence with age being by far the most important factor accounting for over 40% of explained variance. Moreover, lifestyle, general health-, and OA-related factors had generally similar importance in predicting adherence.

A recent systematic review reported that the rates of adherence to digital self-management interventions for OA are generally over 70% with the highest adherence for app-based interventions [10]. In the present study, we observed an average adherence of around 85% for 3- and 12-month follow-ups, which are close to the highest adherence (88%) reported in previous studies [10]. Albeit it should be noted that around 2000 participants had missing adherence at 3 months meaning that these numbers are an overestimation of actual adherence. If we assume an adherence of 0% for those with missing adherence at 3 months, the average adherence drops to 76% which is still consistent with rates observed in previous studies (over 70%) [10]. This also implies that the actual average 3-month adherence in our sample should be between 76 and 85%. The high adherence rates for digital interventions might be due to the convenience, flexibility, and accessibility advantages of these interventions over face-to-face interventions [31]. Self-selection might be another explanation, as participants in digital interventions are possibly more motivated to take an active role in their disease management and are better informed about the benefits of self-management interventions [10]. In addition, and compared to face-to-face interventions, participants in digital interventions are more regularly monitored, which increases the possibility of the Hawthorne effect, i.e. change in one’s behaviour when being observed [32]. It should be noted that cross-study comparisons of the adherence rates and their influencing factors are complicated due to variability in how adherence is measured [22]. Despite high average adherence rates, around 25–30% of the participants in the present study failed to achieve a desirable level of adherence. This highlights the need for additional measures to stimulate adherence, such as group sessions, peer-monitoring, and reminders to promote adherence [33]. According to the information–motivation–behavioural skills (IMB) model, a commonly used model to explain adherence behaviour, to achieve a behavioural change, one should be: well informed about the behaviour, highly motivated to make necessary behavioural changes, and equipped with required skills to perform a specific health behaviour [34]. While the information aspect of the IMB model is covered by the digital self-management intervention, future attempts to promote adherence among the participants should pay more attention to motivation and skill aspects.

Previous research on predictors of adherence to mobile app-based self-management programmes for OA is limited and mainly investigated adherence to home- or web-based exercise programmes. Results suggested that person-level factors, including sociodemographic characteristics, physical activity, health status, fear of moving, baseline pain/function, and psychological disorders, are associated with adherence [31, 33, 35–39]. While these studies assessed the average associations relying on statistical significance and hence were not directly comparable to the current study, their results are consistent with our findings, suggesting that person-level factors contribute to adherence to self-management interventions for OA.

However, the person-level factors analysed in the present study explained only a very modest portion (4–8%) of variance in adherence. In comparison, studies on non-OA cohorts explained 19–24% of variances in adherence to exercise [40] and 40% of adherence to a physiotherapy clinical guideline [41]. The modest explained variance in the current study suggests the presence of considerable heterogeneity in adherence which cannot be explained by the person-level factors included in the present study. This also suggests that any action to improve adherence to the digital intervention should be universal, rather than targeted at a specific subgroup defined based on the predictors investigated in the present study, but with a scale and intensity that is proportionate to the level of need (in line with “proportionate universalism” concept [42]). Adherence is a multidimensional complex behaviour influenced by many factors [16, 39] and the World Health Organization (WHO) grouped these factors into five categories: patient-related, condition-related, therapy-related, socio-economic, and healthcare system/team [16]. The predictors included in the present study mainly covered socio-economic and condition-related factors which explain in part the modest explained variance of adherence in the study. Therefore, there is a need for identifying and measuring/collecting data on other person-level and environmental factors that can improve the ability to predict adherence. For instance, intention to engage, self-efficacy, self-motivation, previous experience with exercise, and attitude towards exercise have been suggested as important predictors of adherence to home-based physical therapies [39, 43].

Large sample size, up to 12-month follow-up data on adherence, and using dominance/relative weight analyses coupled with bootstrapping to determine the importance of adherence predictors are the main strengths of the current study. We also acknowledge some limitations of our study. Data used in the study are self-reported and hence prone to biases. In particular, while adherence is recorded through log into the mobile app, its completion is reported by the participants, which might be overestimated. Indeed, potential errors in the measurement of adherence and predictors could have contributed to the modest explained variance in the present study. Self-selection into the digital intervention may limit the generalizability of the findings. For instance, the participants in the digital intervention are more highly educated compared with the general OA population (e.g. 56% had a college/university level education compared with 22% reported for persons with knee or hip OA in Southern Sweden [44]). Moreover, around 23% of participants in Swedish face-to-face self-management programme (“Better management of patients with OsteoArthritis”) had < 10 years of education [45], while in our sample only around 8% had < 12 years of education. The initial uptake of the digital self-management intervention requires Swedish literacy and access and ability to use a smartphone which limits the generalizability further. For most participants enrolled during the year 2021, the missing 12-month adherence was expected due to the study time frame. However, among those with 3-month adherence data enrolled during 2019–2020 (*n* = 4591), about 52% (*n* = 2393) did not report 12-month adherence. Although the baseline characteristics of respondents and non-respondents were generally comparable, there were some differences in terms of educational attainment (less non-respondents with college/university education), index joint (more non-respondents with hip OA), wish for surgery (higher proportion among non-respondents), and PASS (a lower proportion with “yes” answer among non-respondents). These differences call for caution in interpreting and generalizing the results for 12-month adherence. We observed high level of adherence over 12 months among the participants which might limit the generalizability to population/setting with low level of adherence. The digital intervention is covered by healthcare system in Sweden, and hence, the results might not be applicable to countries with different healthcare and insurance systems. Due to lack of data, we were not able to assess the importance of some potential determinants of adherence including self-efficacy, self-motivation, participants’ perception of OA and its management, social support, physiotherapist characteristics, quality of participant-physiotherapist interactions, and concurrent treatments (e.g. medications) [39, 43]. These unmeasured predictors may contribute to the unexplained variance and should be explored in future studies.

## Conclusions

The findings of this study suggested that while person-level factors were associated with 3- and 12-month adherence to a digital self-management intervention for OA, these factors could only modestly explain the variations in adherence, with sociodemographic characteristics, mainly age, accounting for the greatest portion of the explained variance. These findings highlight the need for identifying other factors that influence adherence to the digital intervention, prior to developing targeted interventions to promote adherence in this population.

## Supplementary Information

**Additional file 1. Table S1.** Associations between predictors and 3-month adherence, estimates obtained from ordinary least square regression. **Table S2.** Associations between predictors and 3-month low adherence (adherence < 80), estimates obtained from logistic regression. **Table S3.**Associations between predictors and 12-month adherence, estimates obtained from ordinary least square regression. **Table S4.** Associations between predictors and 12-month low adherence (adherence < 80), estimates obtained from logistic regression.

## Data Availability

The data that support the findings of this study are available from Joint Academy® but restrictions apply to the availability of these data, which were used under ethical permission for the current study, and so are not publicly available. Data may be made available through the corresponding author upon reasonable request and with permission of Joint Academy®.

## Abbreviations

BMI: Body mass index
CI: Confidence interval
EQ-5D-5L: The 5-level EuroQol-5 Dimensions
OA: Osteoarthritis
NRS: Numerical rating scale
PASS: Patient Acceptable Symptom State
WPAI–OA: Work Productivity and Activity Impairment–Osteoarthritis
